# Development of Cephradine-Loaded Gelatin/Polyvinyl Alcohol Electrospun Nanofibers for Effective Diabetic Wound Healing: In-Vitro and In-Vivo Assessments

**DOI:** 10.3390/pharmaceutics13030349

**Published:** 2021-03-07

**Authors:** Anam Razzaq, Zaheer Ullah Khan, Aasim Saeed, Kiramat Ali Shah, Naveed Ullah Khan, Bouzid Menaa, Haroon Iqbal, Farid Menaa

**Affiliations:** 1 College of Pharmaceutical Sciences, Soochow University, Suzhou 215123, China; 20187126002@stu.suda.edu.cn (A.R.); 20177126002@stu.suda.edu.cn (K.A.S.); 20177226001@stu.suda.edu.cn (N.U.K.); 2 Department of Pharmacy, COMSATS Institute of Information and Technology, Abbottabad 22060, Pakistan; fa19-ppy-003@cuiatd.edu.pk; 3 Collaborative Innovation Center of Advanced Microstructures, School of Chemistry and Chemical Engineering, Nanjing University, Nanjing 210023, China; asaeed@smail.nju.edu.cn; 4 Department of Nanomedicine and Advanced Technologies, California Innovations Corporation, San Diego, CA 92037, USA; bmenaa@cic.com

**Keywords:** gelatin/polyvinyl alcohol, cephradine, diabetic wound, *Staphylococcus aureus*, electrospun nanofibers, translational medicine

## Abstract

Diabetic wound infections caused by conventional antibiotic-resistant *Staphylococcus aureus* strains are fast emerging, leading to life-threatening situations (e.g., high costs, morbidity, and mortality) associated with delayed healing and chronic inflammation. Electrospinning is one of the most widely used techniques for the fabrication of nanofibers (NFs), induced by a high voltage applied to a drug-loaded polymer solution. Particular attention is given to electrospun NFs for pharmaceutical applications (e.g., original drug delivery systems) and tissue regeneration (e.g., as tissue scaffolds). However, there is a paucity of reports related to their application in diabetic wound infections. Therefore, we prepared eco-friendly, biodegradable, low-immunogenic, and biocompatible gelatin (GEL)/polyvinyl alcohol (PVA) electrospun NFs (BNFs), in which we loaded the broad-spectrum antibiotic cephradine (Ceph). The resulting drug-loaded NFs (LNFs) were characterized physically using ultraviolet-visible (UV-Vis) spectrophotometry (for drug loading capacity (LC), drug encapsulation efficiency (EE), and drug release kinetics determination), thermogravimetric analysis (TGA) (for thermostability evaluation), scanning electron microscopy (SEM) (for surface morphology analysis), and Fourier-transform infrared spectroscopy (FTIR) (for functional group identification). LNFs were further characterized biologically by in-vitro assessment of their potency against *S. aureus* clinical strains (*N* = 16) using the Kirby–Bauer test and 3-(4,5-dimethylthiazol-2-yl)-2,5-diphenyltetrazolium bromide (MTT) assay, by *ex-vivo* assessment to evaluate their cytotoxicity against primary human epidermal keratinocytes using MTT assay, and by in-vivo assessment to estimate their diabetic chronic wound-healing efficiency using NcZ10 diabetic/obese mice (*N* = 18). Thin and uniform NFs with a smooth surface and standard size (<400 nm) were observed by SEM at the optimized 5:5 (GEL:PVA) volumetric ratio. FTIR analyses confirmed the drug loading into BNFs. Compared to free Ceph, LNFs were significantly more thermostable and exhibited sustained/controlled Ceph release. LNFs also exerted a significantly stronger antibacterial activity both in-vitro and in-vivo. LNFs were significantly safer and more efficient for bacterial clearance-induced faster chronic wound healing. LNF-based therapy could be employed as a valuable dressing material to heal *S. aureus*-induced chronic wounds in diabetic subjects.

## 1. Introduction

Diabetes is a noncommunicable chronic metabolic disease characterized by elevated levels of blood glucose (hyperglycemia), which over time leads to serious organ damage (e.g., heart, blood vessels, eyes, kidneys, and nerves). The most common is type 2 diabetes (T2D), usually in adults, which occurs when the body becomes resistant to insulin or does not make enough insulin. Type 1 diabetes, once known as juvenile diabetes or insulin-dependent diabetes, is a chronic condition in which the pancreas produces little or no insulin by itself. About 422 million people worldwide have diabetes, the majority living in low-and middle-income countries, and 1.6 million deaths are directly attributed to diabetes each year. Both the number of cases and the prevalence of diabetes have been steadily increasing over the past few decades. There is a globally agreed target to halt the rise in diabetes and obesity by 2025 [1].

Importantly, more than 20% of the diabetic population develops wounds, most commonly in the legs and feet [2]. It is worth noting that, in diabetic patients, hyperglycemia results in a poor wound-healing capability, causing a switch from small injuries to chronic wounds [3,4]. External skin wounds (e.g., cuts, injuries, burns, bruises) and internal skin wounds (e.g., ulcers, calluses) are susceptible to bacterial infection [5]. The diabetic wounds may cause serious, life-threatening complications (e.g., cellulitis associated with depression, gangrene, septicaemia, amputation) due to delayed wound healing, ischemia-induced vascular damage, and chronic inflammation [5,6,7,8]. The persistent inflammatory phase is associated with an impediment in the formation of mature granulation tissue and reduction in wound tensile strength [5]. The impaired diabetic wound healing is influenced by multiple factors including a deficient immune system, poor circulation, disturbances in the metabolic system, infection inclination, and neuropathy resulting in a loss of sensation in diabetic patients [4]. 

Recent studies aimed to develop therapeutics that will promote proper tissue repair and improve impaired wound healing. In this regard, new therapeutic formulations using synthetic and/or natural polymeric materials have been reported, but there is still a paucity of such reports in relation to diabetic skin wounds [9,10,11].

Cephradine (Ceph) is a first-generation cephalosporin antibiotic commonly prescribed intravenously in surgical prophylaxis, pre-operative, and post-operative procedures to prevent the infection and spreading of wounds [12,13]. Despite its relative safety (i.e., no severe side effects in patients not allergic to some antibiotics (e.g., penicillin), except for nausea, diarrhea, seizure, flu symptoms, and unusual bleeding), Ceph is potent (particularly against Gram-positive bacteria), but the emerging resistance to Ceph developed by several bacterial species, including clinical isolates of the Gram-positive *S. aureus*, remains the main challenging issue [14,15,16]. Hence, there is an urgent need to develop advanced Ceph formulations, including for topical application, to better management acute and chronic wounds [12,13,17].

Herein, we decided to investigate the outcomes (in terms of efficiency, safety, rapidity) of newly developed electrospun Ceph-loaded GEL/PVA NFs, abbreviated as LNFs, for diabetic skin wound healing.

Electrospinning is considered the gold standard method to produce NFs [18,19,20,21,22,23,24]. Thereby, in tissue engineering and tissue regeneration, electrospun NFs have gained much interest due to their large surface-to-volume ratio, high porosity, unique ability to mimic the fibrous component of the natural extracellular matrix (ECM), and suitable cell adsorption, adhesion, and proliferation [19,20,21,22,23,24,25,26]. In addition, due to their interconnected pores, sustained/controlled release profile, and high drug-loading capability [24,27], electrospun NFs could be ideal for Ceph delivery.

GEL is a biocompatible, biodegradable, low-immunogenic, and cost-effective biopolymer that has countless applications in the pharmaceutical industry (e.g., development of drug delivery nanocarriers, wound-healing dressings) [18,19,20]. GEL is a nontoxic natural biomacromolecule comprised of bioactive polypeptides derived from collagen (found in animal skin, bones, and connective tissues) by partial hydrolysis [20]. Hence, the capacity of GEL to increase cell adhesion and proliferation subsequently contributes to faster wound healing [20]. NFs represent nanofibrous scaffolds that can serve as substrates for adhesion, growth, and differentiation of skin and stem cells. In addition to their role as an antimicrobial and moisture-retaining barrier, GEL NFs represent a judicious option for wound healing of the skin, the largest vital organ of the human body [20]. Unlike other polymeric systems, GEL electrospun NFs impart controllable thickness and physical stability for various applications such as sustained and controlled drug delivery [24,27,28], in addition to tissue engineering for wound repair (e.g., as a wound-dressing material) [19,20,21,22,23,24,25,26,29].

PVA is a non-toxic, water-soluble synthetic polymer that acts as a glue during electrospinning, subsequently enhancing the mechanical properties of electrospun NFs (e.g., GEL NFs) [30,31,32,33]. Indeed, PVA has good mechanical stability but poor cell adhesion due to the low affinity of protein [34]. It is important to incorporate GEL into PVA solution to form stable electrospun GEL/PVA NFs for their use as wound dressings, considering the unmet medical needs of conventional dressings to completely heal diabetic wounds [23]. In recent studies, Perez-Puyana et al. (2018) investigated electrospun GEL/PVA NFs for tissue engineering [32], and Ahlawat et al. (2019) confirmed the importance of using electrospun GEL/PVA NFs for potential application in wound dressing [33].

Considering the above-mentioned features of GEL, PVA, Ceph, and the electrospinning method, it was worth developing and evaluating LNFs both as a potential antimicrobial delivery system and as a material dressing for accelerated diabetic wound healing. With the aim to reach this important goal, we prepared and characterized electrospun LNFs both in-vitro and in-vivo in an environmentally friendly manner.

To the best of our knowledge, this is the first study reporting the development and testing of LNFs for both diabetic wound infection and healing.

## 2. Materials and Methods

### 2.1. Reagents, Drugs, Cells, and Instruments

PVA (MW: 146–186 kDa, hydrolysis degree: 99+%), gelatin (gel strength~225 g Bloom, Type B), Dimethyl sulfoxide (DMSO), MTT Assay Kit, Muller-Hinton agar (MHA), Muller-Hinton broth (MHB), nutrient agar (NA), and Dulbecco’s Modified Eagle Medium (DMEM) were all purchased from Sigma–Aldrich, Seoul, Korea. Ceph was provided by Bio-Labs Pharmaceutical, Islamabad, Pakistan. *S. aureus* strains (*N* = 16 from 24 diabetic patients) and primary cultures of human epidermal keratinocytes were kindly gifted from hospital wards.

A thermostatic incubator (model: DHP-9052), high-speed refrigerated centrifuge (model: TGL20MC), and multifrequency ultrasonic cleaning machine (model: NB-600 DTY) were purchased from Zhengzhou Nanbei Instrument Equipment Co. Ltd., Zhengzhou, Henan, China. Sample stirring and heating were carried out with a heating magnetic stirrer (model: IKA-RCT-B) purchased from IKA group, Guangzhou, China. A microplate reader (model: Infinite 200 Pro) was purchased from Tecan Trading AG., Männedorf, Switzerland. A thermogravimeter (model: Thermo-plus TG 8120) was purchased from Rigaku Corp., Tokyo, Japan. An SEM (model: Quanta 450 FEG) was purchased from Thermo–Fisher Scientific Inc., San Diego, CA, USA. A UV–Vis spectrophotometer (model: Lambda 950) was purchased from PerkinElmer, Beaconsfield, Buckinghamshire, UK. An FTIR instrument (model: FTIR-4100) was purchased JASCO Inc., Easton, PA, USA.

### 2.2. Synthesis of LNFs

LNFs were fabricated by electrospinning, without using a cross-linking agent, following a similar approach previously reported [30].

Briefly, homogenous GEL solution (50 mg/mL) was prepared in distilled water (D/W) under constant magnetic stirring (340 rpm) at 60 °C for 1 h. In parallel, PVA (50 mg/mL) was dissolved in D/W under continuous magnetic stirring (340 rpm) at 80 °C for 5 h. The PVA solution was then added to the GEL solution at different volumetric ratios of GEL:PVA (1:9, 3:7, 5:5, 7:3, and 9:1), and the mixture was stirred (340 rpm) at room temperature (RT) for 30 min to achieve a homogenous solution. The blank GEL/PVA electrospun NFs (BNFs) were fabricated by transferring each GEL/PVA blend preparation into a 5-mL syringe with a blunt-end 22 G needle. Each GEL/PVA blend solution was then ejected at a feeding/flow rate of 1 mL/hr using the infusion pump NE-300 (New Era Pump Systems Inc., Farmingdale, NY, USA). The distance between the needle tips to the grounded aluminium collector was kept constant at 14 cm. A high voltage of 30 kV was applied to the needle at a relative humidity of 50–60%.

Under an optimized volumetric ratio (5:5 GEL:PVA), LNFs were fabricated by adding Ceph (3% *w/v*) to the PVA solution under continuous magnetic stirring (340 rpm) at room temperature (25 °C) for 30 min, followed by the addition of the GEL solution. The blend was subjected to the same electrospinning process as described for BNFs. The resulting electrospun NFs (BNFs and LNFs) were washed several times with D/W at room temperature to carefully remove any remaining unentrapped drug. They were eventually dried overnight and stored in a desiccator prior to analyses [33]. It is worth mentioning here that such washes could not be recommended if the PVA has a hydrolysis degree of ~87–89%, since this type of PVA dissolves in water at room temperature. Indeed, PVA is a water-soluble polymer, and the dissolution of PVA is essential to form physical hydrogels. However, the dissolution of PVA in water is dependent on the molecular weight and the degree of hydrolysis of the polymer [35]. It has been reported that high dissolution temperatures are required to dissolve PVA in water, especially if it has a high degree of hydrolysis [36,37], due to the strong intra- and intermolecular hydrogen bonds [35,36,37]. PVA with a hydrolysis degree between 85% and 89% dissolves easily in water at RT, while PVA with a high hydrolysis degree (98–99+%) is not water-soluble at RT and can dissolve at temperatures between 80 °C and 90 °C [35]. GEL is completely soluble in water, but only at temperatures above 35–40 °C [38]. At lower temperatures, GEL will swell, rapidly absorbing 5–10 times its weight in water [38]. Upon warming, the swollen GEL will readily dissolve to form a viscous solution [38]. Based on our own observations, and as expected, GEL/PVA blends were not water-soluble at RT; hence, the washing of the electrospun NFs (i.e., BNFs and LNFs) performed with D/W at this temperature. Besides, we found that LNFs were soluble in slightly acidic (0.5% acetic acid) aqueous solution (data not shown).

### 2.3. Determination of Ceph-Loading Capacity and Entrapment Efficiency into BNFs

A specific amount of LNFs (10 mg) was dissolved in 1 mL of slightly acidic (0.5% acetic acid) aqueous solution to extract Ceph from the nanofiber mats and form a solution. After 24 h, a 1:10 dilution of the solution was made in D/W and analyzed for the total Ceph content by spectrophotometry at a wavelength of 256 nm (UV) using a standard curve.

The percent rate of the Ceph-loading capacity (LC) and encapsulation efficiency (EE) were determined as follows:

Ceph LC (%) = (Amount of Ceph in NFs)/(Weight of NFs) × 100

Ceph EE (%) = (Amount of Ceph in NFs)/(Total weight of added Ceph) × 100

### 2.4. SEM Analysis of LNFs

For microstructure analysis and surface fiber morphology, SEM of LNFs was performed following a protocol previously published [30]. BNFs were used as the control: 1/1 cm of each sample was coated by sputtering with a thin layer of gold (Emitech K450X, Ashford, UK). The morphology and average diameter of both LNFs and BNFs were depicted under SEM operated at 10 kV (acceleration voltage).

The micrographs were analysed by using the ImageJ open-source image processing program (US National Institute of Health, Bethesda, MD, USA).

### 2.5. FTIR Analysis of LNFs

The presence of specific functional chemical groups and their structural interactions in fabricated samples were examined by FTIR spectroscopy [30]. The LNFs were separately milled and mixed at a ratio of 1.0% (1 mg dried sample/100 mg KBr) with KBr powder (infrared grade) that was previously dried at 120 °C for 24 h. We did use slow heating since rapid heating (<3 h) could have oxidized some of the KBr powder to KBrO_3_. Then, they were pelletized under vacuum. BNFs and free Ceph were used as controls.

Eventually, the pellets were analyzed in the infrared spectral region of 600–4000 cm^−1^, with 120 scans averaging 4 cm^−1^ resolution, at a scanning speed of 2 mm/s.

### 2.6. Thermostability of LNFs

The thermal stability of the LNFs was evaluated by TGA, following a method previously described [26]. BNFs and free Ceph were used as controls. The same quantity (about 10 mg) of LNFs, BNFs, and free Ceph was heated from 0 to 1000 °C under a constant nitrogen (N_2_) atmosphere at a purge rate of 30 mL/min and a constant rate of 10 °C/min.

The loss in weight (%) observed in the different decomposition steps of the LNF thermogram was compared to that of BNFs and the free drug.

The curve fitting was performed by using Origin Pro 8.0.

### 2.7. Ceph Drug-Release Kinetics from LNFs

The Ceph release behavior from GEL/PVA NFs was studied at pH 7.4 and pH 4.8 using UV–Vis spectrometry at a wavelength of 256 nm. These two pH values (7.4 and 4.8) were chosen to understand the drug release kinetics in the case where LNFs would be intravenously injected or topically applied, respectively. Indeed, pH 7.4 represents the strictly normal blood pH for mammals (e.g., humans and mice). pH 4.8, which is mildly acidic, represents the normal average skin pH for humans and young mice.

Concisely, 10 mg of LNFs was placed inside a dialysis bag which was pre-filled with 5 mL of 1× PBS (adjusted at pH 7.4 or pH 4.8, and thermostated at 37 °C). This temperature was chosen based on the normal human body temperature which is also suitable for experiments in mice (since the mouse body temperature ranges from 36.5 to 38 °C). The dialysis bag was then hung inside a beaker containing 20 mL of the same buffer, which was shaken (150 rpm) at 37 °C for 24 h. Free Ceph (2 mg/mL) was used as a control; 3 mL of the samples was collected at specific time points from the beaker, which was thereafter replenished with fresh buffer.

Non-linear regression fitting curve fitting was eventually used to obtain the parameters and their uncertainties. The Ceph release kinetics from LNFs were described using the Korsmeyer–Peppas method, following this equation [39]:

M_t_/M_∞_ × 100% = Kt*^n^*

where M_t_ is the cumulative amount of drug released at time t; M_∞_ is the mass of the released drug as time reaches infinity; K (=0.708) is a constant characteristic of the drug-polymer system; *n* is the diffusion exponent suggesting the nature of the release mechanism (Table 1). In the case of LNFs, *n* was found to be insignificantly different (i.e., 0.513 at pH 4.8, and 0.512 at pH 7.4).

### 2.8. In-Vitro Antibacterial Activity of LNFs

The antibacterial activity of LNFs was assessed in-vitro against resistant clinical strains of *S. aureus* (*N* = 16) using both the agar well diffusion method (aka Kirby–Bauer test) and MTT assay, as previously described [30].

Briefly, the sensitivity of *S. aureus* strains to LNFs was first analyzed by broth microdilution to determine the minimal inhibition concentration (MIC) of free Ceph, which served as the MIC reference. Serial dilutions (up to 10 two-fold dilutions) of the Ceph solution (2 mg/mL) were prepared in MHB medium. The final concentrations of Ceph in the prepared tubes were 0, 2, 4, 8, 16, 32, 64, 128, 256, 512, and 1024 μg/mL, to which 10 μL (1 × 10^6^ colony forming units (CFUs)) of each bacterial inoculum was added. The inoculated tubes were eventually incubated at 37 °C for 24 h. The lowest concentration (8 μg/mL) showing no cell growth represented the MIC, which was used for subsequent agar well diffusion and MTT assays. It is worth mentioning that 6 μg/mL, defined as the breakpoint concentration recommended by the European Committee on Antimicrobial Susceptibility Testing (EUCAST) (https://www.eucast.org/ accessed on 1 March 2021), was not a sufficient concentration. Indeed, at 6 μg/mL, only 2 Ceph-sensitive *S. aureus* strains (or 14 Ceph-resistant *S. aureus* strains) were detected out of the 16 tested (data not shown).

In a step further, the zone of inhibition (ZI) of LNFs was measured by agar well diffusion assay. MHA was prepared according to the manufacturer’s instructions, sterilized, and poured into Petri plates. Then, the bacterial suspension, matching the 0.5 McFarland standard (prepared by mixing 0.05 mL of 1.175% barium chloride dihydrate (BaCl_2_·2H_2_O) with 9.95 mL of 1% sulfuric acid (H_2_SO_4_)), was spread on Petri dishes using sterile cotton swabs. Subsequently, 18 mm wells were made in the dishes and sealed. Suspensions (100 µL) of LNFs (Test, 8 μg/mL), BNFs (internal control 1, 8 μg/mL), free Ceph (external positive control, 8 μg/mL), or sterilized D/W (external negative control, 100 µL), were added to each well, separately. Then, the plates were incubated at 37 °C for 24 h before examination and measurement of the respective ZIs.

Furthermore, the effect of LNFs on bacterial cell viability was examined by MTT assay through half-maximal inhibitory concentration (IC_50_) determination. In this case, IC_50_ measured the potency of LNFs in inhibiting resistant *S. aureus* cells, compared to that of BNFs and free Ceph, used as controls. Each *S. aureus* strain was cultured in a 100 mm petri dish containing MHA (30 mL). After incubation overnight at 37 °C, 10 μL (5 × 10^5^ CFUs) of each bacterial strain suspension was prepared in sterilized D/W by comparison with the 0.5 McFarland (turbidity) standard against black and white backgrounds. Each bacterial suspension was added to each well of plates containing MHA (100 μL) and incubated for 24 h. After incubation, MHA was replaced with fresh 100 μL MHA containing LNFs and Ceph (50 µL) in each well, separately. Sterilized D/W was used as a control. A range of concentrations (0–128 μg/mL) of LNFs, BNFs, and Ceph was used for this assay. The microplates were incubated at 37 °C for 12 h. Subsequently, 20 μL of MTT reagent (5 mg/mL) was added to each well, and the microplate was further incubated for 4 h. *S. aureus* cells reduced the MTT to the purple color of formazan. The formazan ring was dissolved by adding 120 μL isopropanol, and the production of formazan in each sample was quantified by measuring its absorption at 490 nm using a microplate reader. The absorption was directly related to the number of viable bacterial cells.

The percentage viability of *S. aureus* cells was calculated as follows:

% of Bacterial Cell Viability = (Absorption of sample)/(Absorption of control) × 100

where sample represents LNFs, or BNFs, or Ceph, and control represents D/W.

### 2.9. Ex-Vivo Cytotoxic Evaluation of LNFs

The potential cytotoxic effects of LNFs were evaluated using standard MTT assay [23].

Briefly, various concentrations (range: 0–128 mg/mL) of LNFs were tested on primary cultures of proliferating human epidermal keratinocytes. BNFs, free Ceph, and D/W were used as controls; 1 × 10^4^ keratinocyte cells were cultured in each well of 96-well plates containing 90% DMEM supplemented with 10% fetal bovine serum (FBS) for 24 h. Then, LNFs, BNFs, free Ceph, and D/W were added separately, and the plates were incubated at 37 °C for 24 h. Subsequently, the cells were washed with 1× PBS, treated with MTT reagent, 20 µL (5 mg/mL), and further incubated at 37 °C for 4 h after adding 100 µL of fresh cell culture medium (DMEM/FBS). Eventually, DMSO (150 µL) was added to dissolve the formazan crystals, and the absorption was measured at a wavelength of 490 nm.

The percentage viability of keratinocytes was calculated as follows:

% of Keratinocyte Viability = (Absorption of sample)/(Absorption of control) ×100

### 2.10. Animals and Ethics

Mouse strain NONcNZO10/LtJ, hereafter abbreviated as NcZ10, considered an emerging model of polygenic, moderate type 2 diabetes (T2D) (https://www.jax.org/strain/004456 accessed on 1 March 2021), was used as a model for diabetic wounds. Twenty NcZ10 male mice 8–10 weeks old, purchased from the Department of Experimental Animals, Soochow University, China, were maintained under standard controlled housing conditions. NcZ10 mice were maintained on high-fat diet. All animal experiments were carried out in accordance with guidelines evaluated and approved by the ethics committee of Soochow University, China (No. 2019LW003).

### 2.11. In-Vivo Wound Healing Efficiency of LNFs

NcZ10 diabetic and obese mice (*n* = 18) aged 12–14 weeks with an average body weight of 60 g were used for the development of chronic wounds.

All mice were shaved before a depilatory lotion was applied (to obtain a smooth skin). Buprenex (0.05 mg/kg mouse in 1× PBS) was injected intraperitoneally for pain relief 30 min before surgery and 6 h after surgery. The wound regdox agent Inject 3-amino-1,2,4-triazole (ATZ) (1 g/kg mouse in 1x PBS), was also injected intraperitoneally 20 min before surgery. A wound was created in 30–45 s using a 7 mm skin biopsy punch, tweezers, and surgical scissors. The biopsy punch was lightly pressed onto the anticipated wound location before it warped. The outlined skin was expunged by dragging up the punch centre and cutting it along the outline with surgical scissors. Only one wound developed in each mouse (*n* = 18) because mice can only withstand the burden of one wound. Then, 9 × 10^8^ CFUs of a Ceph-resistant *S. aureus* suspension (100 μL) were injected into the local wound area of each mouse. After producing infected wounds in the diabetic mice, the mice were divided into three groups (*n* = 6/group): Group 1: mice were treated with LNFs, Group 2: mice were treated with free Ceph, Group 3: mice were treated with BNFs. LNFs, BNFs, or free Ceph was applied topically (to the wound area) on a daily basis for 11 days. The wound was then covered firmly with a sterilized transparent dressing film (5 × 5 mm^2^). The wound area of each mouse was measured by a digital vernier caliper on days 1, 7, and 11 after bacterial inoculation [4]. The wound closures on these days were reported in ratio-based calculations relative to the first day wound (100%, day 1) and were compared between the three mice groups.

Furthermore, the eventual bacterial clearance from the diabetic wound was validated by the colony counting method [40]. Briefly, the homogenized wound area tissue sample of each mouse (*n* = 18) was serially diluted (4-fold dilution) for bacterial counts. Then, 100 µL of the homogenized tissue was added to each NA plate and incubated at 37 °C for 24 h for potential bacterial growth. After incubation, the number of bacterial colonies was estimated by using a colony counter, and the CFU was calculated by the following formula:

$$\mathrm{CFU}/\mathrm{mL}=\frac{Number\;of\;colonies\;\times Dilution\;factor}{Volume\;of\;culture\;plate}$$

Subsequently, all the experimental mice were sacrificed, by cervical dislocation, at the end of the study. Cervical dislocation is a common and appropriate method of euthanasia for mice and small rats (<200 g) since it is largely assumed that it relieves pain and suffering.

### 2.12. Statistical Analysis

All experiments were performed at least in triplicate. All data are expressed as the mean ± standard deviation.

The statistical analysis of differences was performed using a t-test in OriginPro 2018.

*p* < 0.05 was considered to indicate a statistically significant difference.

## 3. Results and Discussion

Ceph, a broad-spectrum first-generation cephalosphorin antibiotic, was proved to be effective against soft tissue infections and wounds, but the emerging *S. aureus* resistance to Ceph constitutes a challenging issue, including for diabetic wound healing [11,12,13].

In the present study, we developed an innovative biocompatible delivery system to heal chronic diabetic wounds by sustained and effective Ceph delivery.

### 3.1. Green Preparation of Electrospun LNFs

Unlike other synthesis protocols, electrospinning is a popular method for generating continuous elongated NFs (at a high aspect ratio and a high degree of fiber orientation) due to its simplicity and versatility [30,41]. In various fields (e.g., tissue engineering, tissue regeneration, wound healing, as well as in drug delivery), electrospun NFs attract much attention because of their interesting and unique features (e.g., fascinating morphologies exhibited by their self-assembly capacity, ability to mimic the fibrous component of the natural ECM, biocompatibility, good structural stability, high porosity, large surface-to-volume ratio), which subsequently ensure (i) good cell adsorption, adhesion, and proliferation, (ii) high drug payload, (iii) safe controlled release efficiency [19,20,21,22,23,24,25,26,29,30,41].

GEL/PVA blends were prepared in D/W at different volumetric ratios (i.e., 1:9, 3:7, 5:5, 7:3, and 9:1), with or without Ceph. LNFs were ecofriendly synthesized by electrospinning (without the use of a chemical cross-linking agent). Optimized LNFs were fabricated with Ceph (3% *w/v*) and GEL:PVA (5:5, *v/v*). Interestingly, % LC and% EE were found to be 11.4% and 63%, respectively. These data are encouraging, based on our previous study [30], that reported% LC of 10.3% and % EE of 51.5% when another antibiotic, Cefadroxil, was loaded into electrospun chitosan (CS)/PVA NFs as an effective antibiotic nanocarrier system to fight resistant *S. aureus* strains-induced wound infection. These data also support the fabrication of electrospun GEL/PVA NFs without the use of a (chemical or physical) cross-linking agent [30].

The success of LNF synthesis was further checked based on morphological characteristics, thermostability, swelling behavior, safety, and efficiency.

### 3.2. Physical Characterization of Electrospun LNFs

**Figure 1:**
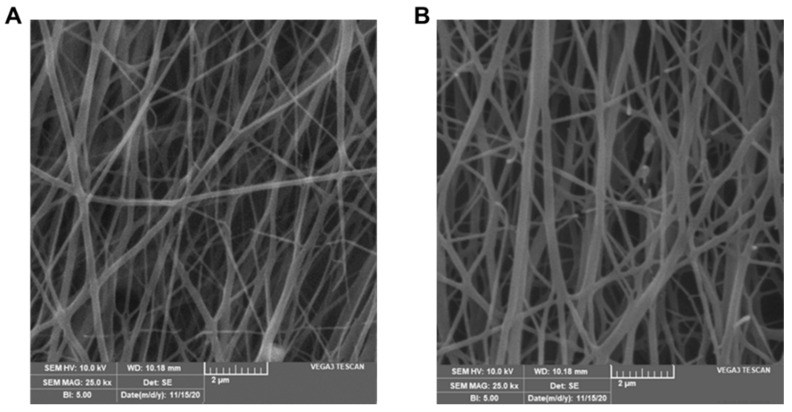
SEM micrographs of (**A**) BNFs and (**B**) LNFs prepared at a 5:5 GEL:PVA volumetric ratio.

**Figure 2:**
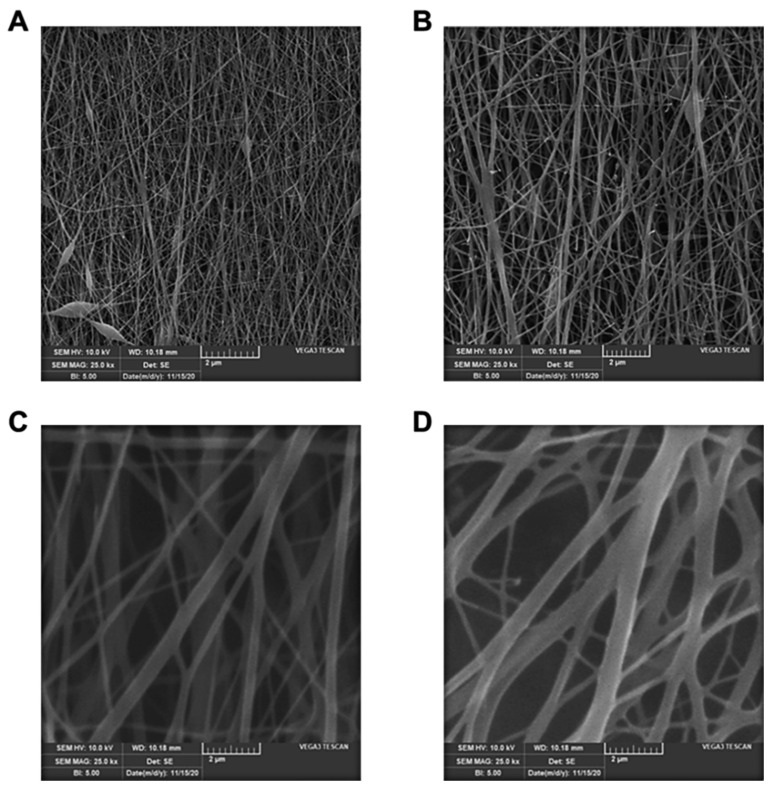
SEM micrographs of LNFs prepared at different GEL:PVA volumetric ratios. (**A**) 9:1; (**B**) 7:3; (**C**) 3:7; (**D**) 1:9.

The morphology of LNFs and BNFs was evaluated by SEM at 10 kV and 25× magnification. SEM micrographs of all blended NFs formulations were compared (Figure 1 and Figure 2). Interestingly, we found that both LNFs and BNFs, prepared at the optimized 5:5 (GEL:PVA) volumetric ratio, exhibited the finest uniformity resembling natural fibers. This observation suggests that adding Ceph (Figure 1B) did not alter the morphological aspect of the BNFs (Figure 1A).

It is worth mentioning that electrospun thin fiber uniformity is extremely favoured in wound healing and skin tissue regeneration [19,20,21,22,23,24,25,26,29,30,41]. In addition to their flush homogeneity, the surface of electrospun NFs was smooth with no interface layer appearance and no pores visible at 25× magnification (Figure 1).

The average diameter of LNFs was 350 ± 20 nm (Figure 1B), which was significantly higher (*p* < 0.05) compared to that of BNFs, with 285 ± 32 nm (Figure 1A). This noticeable difference is most likely due to drug loading-related viscosity changes. Indeed, increased viscosity and subsequent variations of NF diameter upon loading antibacterial drugs can be explained by stress relaxation time changes and chain exertion of the synthetic polymer PVA [28,30]. Thereby, by increasing the volumetric ratio of PVA, the viscosity of the GEL/PVA blend is known to increase while its conductivity decreases, leading to decreases in the surface charge density, reduced repulsive force of the jet, and larger diameter of NFs [28,30]. In accordance with that statement, we clearly noticed that increasing the PVA volume in GEL/PVA formulations (i.e., 3:7, 1:9, *v/v*) resulted in a significant increase (*p* < 0.05) in the average diameter of the electrospun LNFs (Figure 2C,D) compared to that of electrospun LNFs prepared with GEL/PVA at the 5:5 volumetric ratio (Figure 1B). Conversely, a decrease in PVA volume in GEL/PVA formulations (i.e., 9:1, 7:3, *v/v*) resulted in a significant decrease (*p* < 0.05) of the average diameter of electrospun LNFs (Figure 2A,B) when compared to electrospun LNFs prepared with GEL/PVA at the 5:5 volumetric ratio (Figure 1B).

Therefore, the optimized volumetric ratio of the GEL/PVA blend was set to 5:5.

**Figure 3:**
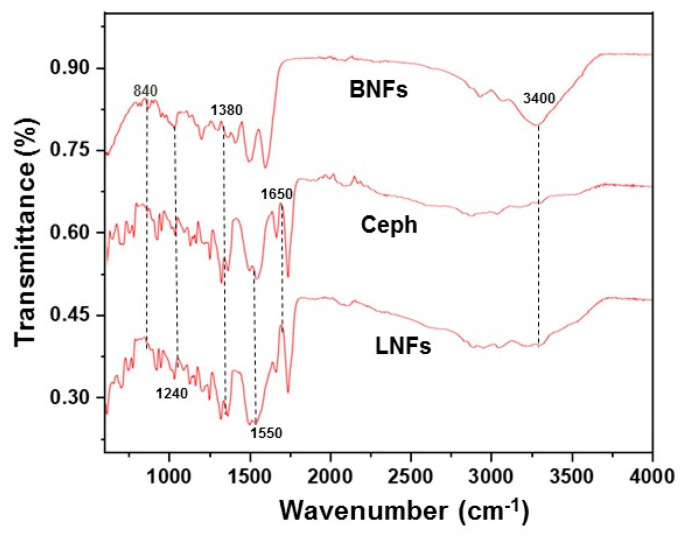
FTIR spectrum of LNFs, BNFs, and Ceph.

Further, the successful drug loading into BNFs was confirmed by FTIR spectroscopic analyses recorded in the spectral range of 600–4000 cm^−1^. As depicted in Figure 3, differences were observable between BNFs and LNFs, not only in terms of spectral absorbances (BNFs > LNFs), but also in terms of spectral patterns (i.e., slight shifts in characteristic peaks). These observations indicate that bonding strongly occurred between the drug and the GEL/PVA blended polymers.

Briefly, the absorption band at 3400 cm^−^^1^ in BNFs is assigned to N-H stretching vibrations. The bands at 1680 cm^−^^1^ and 1650 cm^−^^1^ in LNFs and free Ceph are specifically attributed to C=O stretching vibration representing amide and lactam classes, respectively. The absorbance bands at 1380 cm^−^^1^ and 1240 cm^−^^1^ are assigned to C-H and N-H bending, respectively [31,32]. In addition, the discrete bands in the range of 1450–1000 cm^−^^1^ (C-H bending and wagging) observed for the nanomaterials are related to CH_2_ symmetrical and asymmetrical stretching [32].

The FTIR profile displayed by LNFs showed a combination of both BNFs and free Ceph spectra (dash lines), confirming Ceph loading into BNFs.

### 3.3. Thermal Stability of LNFs

**Figure 4:**
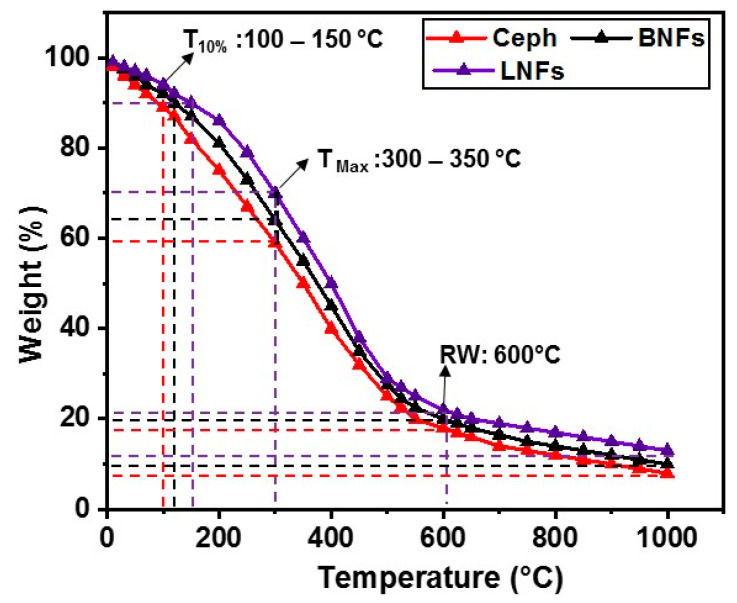
TGA of LNFs, BNFs, and Ceph.

The thermal stability of LNFs was estimated by TGA using BNFs and free Ceph as controls (Figure 4). TGA scans were carried out as described in Section 2.6. A similar protocol was previously reported by our group [30]. TGA is generally considered a three-step process since it exhibits three weight losses with temperature in an inert atmosphere (e.g., N_2_) [42].

Initially, minimal (10%) onset weight loss (WL) was noticed at T_10%_, which was reached at 150 ± 5 °C, 120 ± 3.5 °C, and 100 ± 4.2 °C for LNFs, BNFs, and free Ceph, respectively. This phenomenon is explained by moisture vaporization being significantly higher (*p* < 0.05) in NFs than in the free drug [30]. After loss of moisture, we observed a second WL for LNFs (21 ± 2.0%), BNFs (27 ± 2.7%), and free Ceph (35 ± 3.9%) at T_max_ (325 ± 10 °C). The significant difference (*p* < 0.05) between WL T_max_ for LNFs and BNFs was then contributed by Ceph drug loading. Around 600 °C, the residual weight (RW) was insignificantly different (*p* > 0.05) between LNFs (21 ± 0.8%), BNFs (20 ± 0.6%), and free Ceph (19 ± 0.7%). At the end of the analysis carried out at the highest temperature (1000 °C), all the samples lost most of their water (≥90%), with no significant differences (*p* > 0.05) between LNFs (11 ± 1.3%), BNFs (10 ± 1.1%), and free Ceph (9 ± 0.9%).

Taken together, TGA data showed that LNFs are thermostable to withstand elevated temperatures. Importantly, these data also revealed that free Ceph is more susceptible to thermal instability/degradation compared to LNFs. Similar conclusions were reported by our group [30] and other teams worldwide when electrospun GEL/PVA NFs [32] or polysaccharide/PVA NFs [43] were characterized for potential application in wound dressing and/or compound delivery.

### 3.4. In-Vitro Ceph Release Behaviour from LNFs

**Figure 5:**
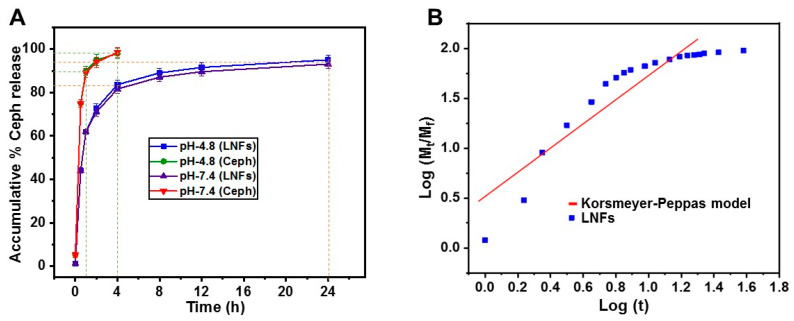
In-vitro Ceph release behaviour from LNFs (in 1× PBS, at 37 °C, for 24 h). (**A**) Ceph release kinetics from LNFs compared to that of free Ceph used as a control; (**B**) Curve fitting using the Korsmeyer–Peppas model (at pH 4.8).

In-vitro Ceph release kinetics from LNFs were evaluated for 24 h by UV–Vis spectrometry in 1× PBS thermostated at normal human/mouse body temperature (37 °C) and adjusted either to normal mammalian blood pH (7.4) or human/young mouse skin surface pH (4.8) (Figure 5A).

After 1 h, the release of free Ceph was about 90 ± 1.25%, whereas the release of Ceph from LNFs was about 60 ± 1.12% (*p* < 0.05). This effect was independent of the pH (*p* > 0.05).

After 4 h, the release of free Ceph was already recorded as maximal (98 ± 1.21%), whereas the release of Ceph from LNFs was about 80 ± 1.95% (*p* < 0.05). This effect was independent of the pH (*p* > 0.05).

After 24 h, the release of Ceph from LNFs was 92 ± 2.23% and 95 ± 1.68% at pH 7.4 and pH 4.8, respectively. This effect was pH-independent (*p* > 0.05).

The pH-independent burst release of Ceph from LNFs recorded within the first 4 h, as well as the pH-independent sustained Ceph release observed in the next 20 h, is highly desired for wound healing/topical administration (pH 4.8) and/or drug delivery/intravenous route (pH 7.4), both in humans and young mice [28,30].

The Ceph release kinetics from LNFs (Figure 5B) were described using the Korsmeyer–Peppas method [39]. According to the release exponent “*n*”, found to be 0.513 at pH 4.8 and 0.512 at pH 7.4 (*p* > 0.05), the release of Ceph from LNFs ensued mainly by diffusion following the non-Fickian (anomalous) transport mechanism (Table 1). The burst release of Ceph from LNFs during the initial period of 4 h, followed by the sustained antibiotic drug release profile (plateau) for the next 20 h, reflected zero-order kinetics (process of constant drug release), which might be due to the hydrophilic nature of PVA [30,39]. It was previously reported that the cross-linked network of such a drug delivery nanosystem decreases the release rate of the encapsulated drug [30]. The present data showed prominent release kinetics of LNFs under physiological-like conditions, which could be applied efficiently as a transdermal Ceph delivery system. Indeed, although the physiological conditions are much more complex than buffer solutions, it is admitted that the in-vitro release results reveal the structure–function relationship of the material matrices, contribute to the tailoring of material for optimal controlled release, and also providing insights into the performance of the formulation in-vivo [44,45].

### 3.5. In-Vitro Antibacterial Activity of LNFs

**Figure 6:**
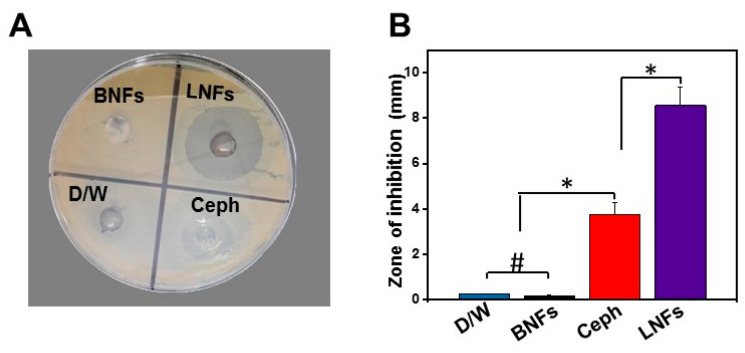
Antibacterial activity of LNFs (8 μg/mL) against clinical *S. aureus* strains (*N* = 16). (**A**) agar-well diffusion assay. Free Ceph at MIC (8 μg/mL), BNFs (8 μg/mL), and D/W (100 μL) were used as a positive control, a potential internal negative control, and an external negative control, respectively; (**B**) Statistical analysis and graphical representation of relative ZIs. Data are expressed as the mean ± SD. * *p* < 0.05, and ^#^ *p* > 0.05, represent statistical significance and statistical insignificance, respectively.

**Figure 7:**
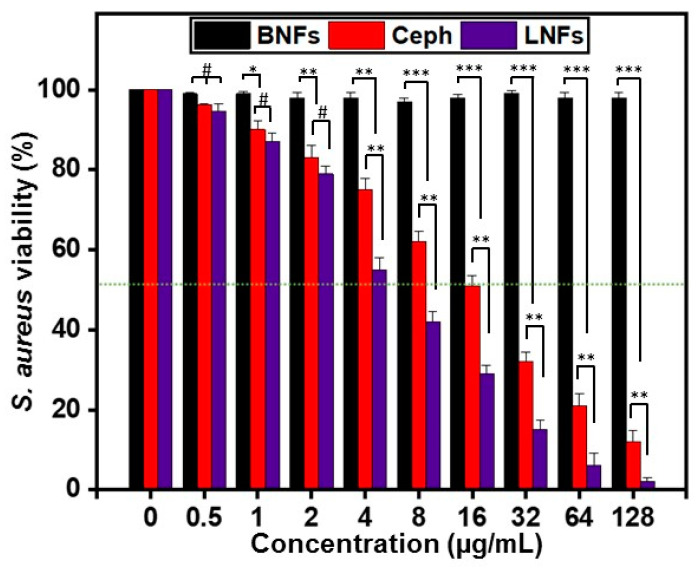
Viability of *S. aureus* strains treated with LNFs at indicated concentrations. Free Ceph and BNFs were used as a positive control and negative control, respectively. Results are expressed as the mean ± SD. *** *p* < 0.001, ** *p* < 0.01, * *p* < 0.05 are differences considered statistically significant, and ^#^ *p* > 0.05 is considered statistically insignificant.

To investigate the antibacterial activity of LNFs against clinical *S. aureus* isolates, the agar-well diffusion method (Figure 6) and MTT assay (Figure 7) were used to determine ZI and IC_50_, respectively.

Among the isolated *S. aureus* strains (*N* = 16), only two (12.5%) strains showed susceptibly to Ceph, while the remaining strains (87.5%, *n* = 14) elicited resistant against the breakpoint value of Ceph (6 μg/mL) defined by EUCAST (https://www.eucast.org/ accessed on 1 March 2021). This finding is in agreement with previous studies. For instance, Zaman et al. reported that *S. aureus* strains are highly resistant to Ceph [46]. Therefore, we determined, by broth microdilution method, the lowest concentration (MIC) of Ceph required to inhibit visible cell growth of the resistant *S. aureus* strains, which was eventually found to be 8 μg/mL.

Dilution methods (e.g., macrodilution or microdilution, agar dilution) are the most appropriate for the determination of MIC values because they offer the possibility to quantitatively estimate the in-vitro antimicrobial (i.e., antibacterial or antifungal) activity of the tested antimicrobial agent in medium [47]. There are many approved guidelines for dilution antimicrobial susceptibility testing, but the most recognized standards are provided by the Clinical and Laboratory Standards Institute (CLSI) and EUCAST [47].

Interestingly, data from agar-well diffusion test (Figure 6A,B) indicated that LNFs at MIC Ceph (8 μg/mL) exerted about three times higher antimicrobial activity against all the tested clinical *S. aureus* strains compared to that of free Ceph (*p* < 0.05). Indeed, ZI averaged 9.56 ± 1.67 mm and 3.23 ± 1.5 mm for LNFs and free Ceph, respectively (Figure 6B). BNFs also used at MIC Ceph did not elicit a detectable inherent antibacterial activity and was then used as an internal negative control. Expectedly, D/W that was used as an external negative control, did not display a detectable ZI.

Agar disk-diffusion testing developed in 1940 is the official method used in many clinical microbiology laboratories for routine antimicrobial susceptibility testing, which can be for drug discovery, epidemiology, and prediction of therapeutic outcomes [47].

Further, the IC_50_ of LNFs against *S. aureus* cells, determined by MTT assay, was compared to that of free Ceph and BNFs, used as a positive control and negative control, respectively.

Importantly, LNFs inhibited bacterial cell growth with an IC_50_ value of 6.005 ± 1.0 µg/mL, which was significantly higher (*p* < 0.01) than that of free Ceph for which the IC_50_ value averaged 16.746 ± 1.2 µg/mL (Figure 7). These data are consistent with the previous antibiotic susceptibility testing we performed by agar-well diffusion assay. They also explained why a high level of *S. aureus* was resistant to free Ceph when 6 μg/mL was used as the breakpoint concentration value.

It can be pointed out that the methodological standards do not guarantee the clinical relevance of such testing, and the importance to perform one’s own bioassays in a standardized approach should be emphasized in order to evaluate the correct MIC and ensure the clinical relevance of results [47].

Overall, the in-vitro data analyses revealed that such an antimicrobial hybrid nanocarrier system is a promising therapeutical option because of its substantially prolonged activities in addition to its capacity to significantly better tackle Ceph resistance compared to that of its free counterpart (free Ceph). In general, antibiotic-loaded NFs show enhanced antibacterial and antifungal activities by inhibiting cell wall synthesis (e.g., Ceph), protein synthesis, DNA/RNA synthesis, mycolic-acid synthesis, and/or folic acid synthesis. A study carried out with ciprofloxacin (Cipro)-loaded electrospun CS NFs, intended to be used as potential antibacterial wound dressing material, reported improved antibacterial activity compared to that of free Cipro when tested against *Escherichia coli*, *Klebsiella pneumonia*, and *Candida albicans* [48]. For the same purpose, our team recently revealed higher antibacterial activity of cefradoxil-loaded electrospun CS/PVA NFs against *S. aureus* clinical isolates compared to that of free cefradoxil [30].

### 3.6. Ex-Vivo Cytotoxic Effects of LNFs

**Figure 8:**
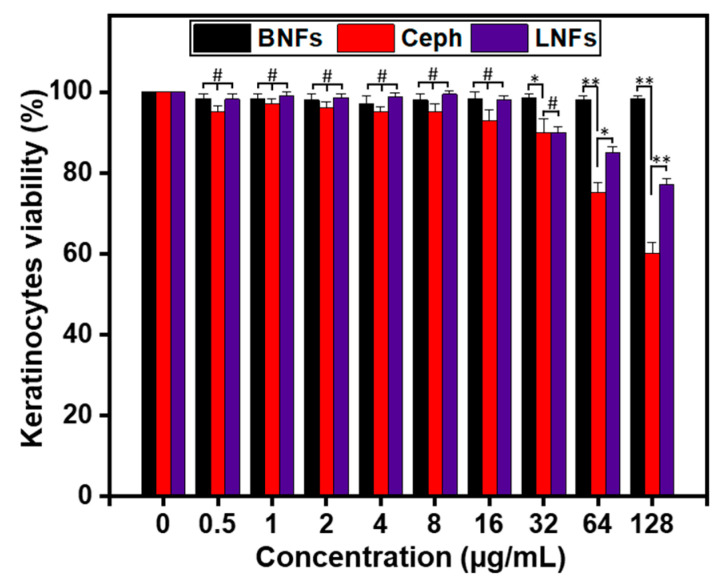
Viability of primary epidermal keratinocytes treated with LNFs at indicated concentrations. BNFs and Ceph were used as controls. Results are expressed as the mean ± SD. ** *p* < 0.01, * *p* < 0.05 are differences considered statistically significant, and ^#^ *p* > 0.05 is considered statistically insignificant.

The potential cytotoxic effects of LNFs were evaluated at various concentrations (range: 0–128 μg/mL) on primary cultures of proliferating human epidermal keratinocytes (1 × 10^4^ cells/well in 96-well plates) using MTT assay (Figure 8). Free Ceph and BNFs were used as controls.

Up to 16 μg/mL, no cytotoxic effect was noticed statistically when the cells were treated either with LNFs or Ceph compared to BNFs (*p* > 0.05).

Nevertheless, from 32 μg/mL, both LNFs and Ceph exhibited significant cytotoxicity compared to BNFs (*p* < 0.05). This cytotoxic effect appeared to be concentration-dependent with the most significant difference noticed at 128 μg/mL (*p* < 0.01).

Furthermore, no statistical differences in cytotoxicity were observed up to 32 μg/mL when the viability rate (%) of LNF-treated cells was compared to that of free Ceph-treated cells (*p* > 0.05).

However, from 64 μg/mL, a significantly higher rate (%) of viable LNF-treated cells was observed compared to that of free Ceph-treated cells (*p* < 0.05).

We postulate that the LNF-induced lower cytotoxicity (compared to that of Ceph) is more likely due to the indirect contact of Ceph with the keratinocytes. Indeed, in LNFs, Ceph was encapsulated into the biocompatible polymer GEL, and no concentration-dependent cytotoxicity was detected with BNFs compared to untreated cells (*p* > 0.05).

Previously, electrospun NFs have been reported for tissue engineering and wound-healing applications [19,20,21,22,23,24,25,26,29,30,41,43,48,49], and some were found to be very potent and safe to combat bacterial infections [24,30]. The low toxic effects of LNFs on skin cells (compared to Ceph) have encouraged testing of LNFs for chronic diabetic wound healing and bacterial clearance in-vivo.

### 3.7. In-Vivo Wound-Healing Efficacy of LNFs

Diabetic wounds are a painful issue with the deterioration of the epidermis, dermis, and subcutaneous tissue [2,5,6]. Chronic wounds are more common in diabetic lower extremities, particularly the foot [2,5]. Microbial invasion is one the major troublesome factors in almost all types of diabetic chronic wounds, causing failure in tissue repair and wound healing [2,5]. Searching for effective and progressive wound-healing strategies for diabetic patients is an unmet prerequisite of the current clinical diagnostics [6,31].

**Figure 9:**
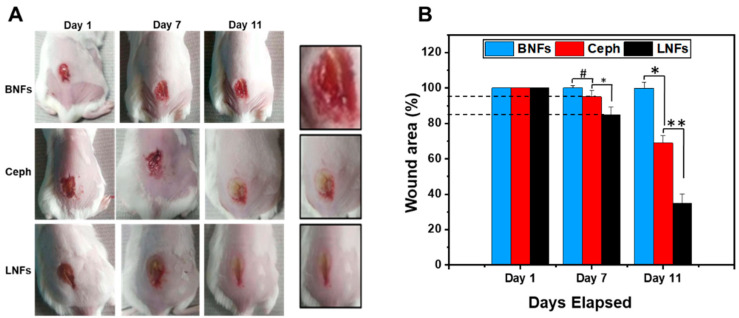
Wound healing capacity of LNFs in the diabetic/obese NcZ10 mouse model. (**A**) One single *S. aureus*-infected wound was created by excision before daily topical treatment with LNFs for 11 days. Ceph and BNFs were used as controls; (**B**) Graphical representation of wound area closure (%) over time in the three groups of mice. Data are expressed as the mean ± SD. ** *p* < 0.01, * *p* < 0.05 are differences considered statistically significant, and ^#^ *p* > 0.05 is considered statistically insignificant.

Therefore, we tested in-vivo LNFs as a sustained antimicrobial delivery nanosystem and tissue/electrospun nanofibrous scaffold to enable bacterial clearance from diabetic chronic wounds while ensuring effective (efficient, safe, and fast) wound healing. For this purpose, we applied the diabetic/obese NcZ10 mouse model in which a single *S. aureus*-infected wound was created by excision. *S. aureus* cells were *injected intracutaneously.* The wound area of each mouse was measured by a digital vernier caliper, and the percent area was calculated. The wound area closure was monitored over time after topical application (directly on the skin surface) of LNFs (Test Group/Group 1) (Figure 9A). Ceph (Group 2) and BNFs (Group 3) were used as controls. Immediately after treatment, each infected wound was recovered with a sterilized transparent dressing film (5 × 5 mm^2^). The percent wound closure was reported for each treated group of mice (six mice per group) on days 1, 7, and 11 (after creating the infected wound), in ratio-based calculations relative to the wound on day 1 (100%) (Figure 9B).

On day 7, the wound healing efficacy of Group 1 was 15 ± 4.2% (or 85 ± 4.2% unclosed wound area), whereas it was significantly lower (*p* < 0.05) in Group 2, with 5 ± 3.5% (or 95 ± 3.5% unclosed wound area). In addition, the wound closure observed in Group 2 was insignificantly different compared to that of Group 3 (*p* > 0.05). The wound healing in Group 3 was non-existent, 0 ± 1.25% (100 ± 1.25% unclosed wound area) and comparable to that observed on day 1 (*p* > 0.05).

On day 11, the wound-healing efficacy in Group 1 was about 65 ± 5.2% (or 35± 5.2% unclosed wound area), whereas it was significantly lower (*p* < 0.01) in Group 2, with 31% ± 4.11% (or 69 ± 4.11% unclosed wound area). This wound healing observed in Group 2 was significantly higher than the effect seen in Group 3 (*p* < 0.05). The wound healing in Group 3 was non-existent, 0 ± 3.04% (99.8 ± 3.04% unclosed wound area) and comparable to that observed on day 1 or 7 (*p* > 0.05).

Although the progressive wound closure was not completed with a microscopic sample for histopathological study, the stages of the wound-healing process were extensively reviewed by Tolotti et al. (2020) [11]. In addition, the wound closure was observed over time by comparative visual analyses [4], and the bacterial clearance on day 11 was confirmed from LNF-treated wounds using the colony counting method [40]. The bacterial counting was performed using the whole wound area taken from each mouse. As reported in Table 2, LNFs completely improved the bacterial clearance elicited by free Ceph (*p* < 0.001), which was significantly better than that elicited by BNFs (*p* < 0.05).

The wound-healing process in diabetic mice was a time-dependent phenomenon. Indeed, topical application of LNFs exerted a greater wound healing compared to that of free Ceph applied under the same experimental conditions, and the greatest wound healing was noticed on day 11. The efficiency of LNF-mediated wound healing appeared to be linked to its capacity to clear bacteria, including the intradermal inoculated resistant *S. aureus* strains. Conversely, although BNFs were found to be safe nanomaterials ex-vivo, they did not exert any benefits alone on wound healing in diabetic mice. Thus, our study demonstrated the high benefit/risk ratio in combining Ceph with BNFs, which would likely act as an adjuvant to improve the outcome obtained with free Ceph to fight resistant *S. aureus*. Our data revealed that LNFs might be efficiently and safely used in clinical practice as a biodegradable, biocompatible, and low immunogenic dressing material for bacterial clearance, especially to overcome resistance associated with *S. aureus*-induced diabetic skin wounds.

To the best of our knowledge, this is the first report that demonstrates the benefits of a topical route for Ceph administration through LNFs, as a dressing material/tissue scaffold, and a sustained drug system delivery. This original nanocarrier could overcome the antibiotic resistance commonly associated with *S. aureus*-induced infection and inflammation. Few recent studies showed possible benefits of using stem cells [6,8], topical drugs formulation [10], and biopolymeric nanocomposites (e.g., unloaded GEL/PCL nanofibrous scaffolds containing silicate-based bioceramic particles) [26], for the treatment of diabetic wounds, but more investigations are needed to comparatively determine the best benefit/risk ratio.

## 4. Conclusions and Perspectives

Currently, there are inadequate clinical data for the bacterial clearance from human diabetic wounds and chronic diabetic wound healing. Most of the monotherapies failed to treat the multifactorial diabetic chronic skin wounds. Furthermore, a lot of work is still needed for the firm understanding of all stages of diabetic wound healing. Diabetes is synonymous with compromised life quality with the risk of deleterious skin ulcerations. Thus, with the ambition of discovering original and accessible therapeutics, we strived to design an eco-friendly and cost-effective therapeutic strategy for effective healing of diabetic skin wounds. Resistant *S. aureus* is present in about one-quarter of diabetic infections and is associated with a higher rate of treatment failure, morbidity, and hospitalization cost.

Ceph-loaded BNFs (LNFs) were rapidly produced in an eco-friendly manner by electrospinning at the optimized GEL:PVA volumetric ratio of 5:5. Ceph, a broad-spectrum cephalosporin antibiotic, is commonly prescribed intravenously in surgical procedures to prevent the infection and spreading of wounds. LNFs were successfully characterized both physically and biologically. Thin and uniform NFs with a smooth surface and standard size (<1000 nm/1 μm) were observed by SEM. FTIR analyses confirmed the drug loading into the (BNFs) nanocarrier. LNFs were significantly more thermostable than free Ceph. Ceph release from LNFs was controlled/sustained through diffusion following a non-Fickian (anomalous) transport mechanism, which was not the case for free Ceph. Further, LNFs were significantly safer than Ceph in primary keratinocytes, which favoured their application on the skin surface. Importantly, LNFs were significantly more effective than Ceph in eradicating resistant *S. aureus* clinical strains both in-vitro and in-vivo. The electrospun LNFs allowed faster wound healing in diabetic/obese NcZ10 mice compared to that of free Ceph, and this appeared to be linked to their bacterial clearance capacity.

Electrospun LNFs could represent a promising therapeutic option, in terms of potency and safety, to prevent both resistant Staph infections and life-threatening complications in diabetic subjects. LNFs exerted a dual function, as a Ceph delivery nanosystem and as a wound-dressing nanomaterial. The WHO recommends a surveillance program for monitoring antibiotic resistance. The present study is a step in this direction.

## Figures and Tables

**Table 1:** Diffusion exponent and respective drug-release mechanism [39].

**Diffusion Exponent (** ***n*** **)**	**Drug-Release Mechanism**
0.5	Fickian diffusion
0.5 > *n* < 1.0	Non- Fickian or anomalous transport
1.0	Case-II transport
>1.0	Super case-II transport

**Table 2:** Number of bacteria from diabetic wounds after specified topical treatment on day 11. ND (Not Detected).

No. Mice	BNFs (Group 3)	Ceph (Group 2)	LNFs (Group 1)
1	1.9 × 10^9^	1.9 × 10^7^	ND
2	1.2 × 10^10^	3.9 × 10^7^	ND
3	2.0 × 10^9^	2.2 × 10^7^	ND
4	1.0 × 10^10^	2.8 × 10^7^	ND
5	1.4 × 10^9^	2.9 × 10^7^	ND
6	1.2 × 10^9^	3.7 × 10^7^	ND
Mean	145 × 10^7^	2.9 × 10^7^	ND
SD	40 × 10^7^	0.7 × 10^7^	ND

## Data Availability

The data presented in this study are available on request from the corresponding authors.
