# Correction: Razzaq et al. Development of Cephradine-Loaded Gelatin/Polyvinyl Alcohol Electrospun Nanofibers for Effective Diabetic Wound Healing: In-Vitro and In-Vivo Assessments. *Pharmaceutics* 2021, *13*, 349

**DOI:** 10.3390/pharmaceutics17010126

**Published:** 2025-01-17

**Authors:** Anam Razzaq, Zaheer Ullah Khan, Aasim Saeed, Kiramat Ali Shah, Naveed Ullah Khan, Bouzid Menaa, Haroon Iqbal, Farid Menaa

**Affiliations:** 1 College of Pharmaceutical Sciences, Soochow University, Suzhou 215123, China; 2 Department of Pharmacy, COMSATS Institute of Information and Technology, Abbottabad 22060, Pakistan; 3 Collaborative Innovation Center of Advanced Microstructures, School of Chemistry and Chemical Engineering, Nanjing University, Nanjing 210023, China; 4 Department of Nanomedicine and Advanced Technologies, California Innovations Corporation, San Diego, CA 92037, USA

In the original publication [1], there was a mistake in Figure 9 as published. While preparing wound images of the in vivo study, one image in Figure 9 for the Ceph-treated group was inadvertently misplaced. The reported issue was only in the inadvertent placement/duplication of one representative wound image in the Ceph-treated group in Figure 9. The corrected Figure 9 appears below.

## Email Update

The corresponding author’s email address has been updated. The updated email information is as follows: 20177226002@stu.suda.edu.cn (H.I.); menaateam@gmail.com (F.M.).

The authors state that the scientific conclusions are unaffected. This correction was approved by the Academic Editor. The original publication has also been updated.

## Figures and Tables

**Figure 9:**
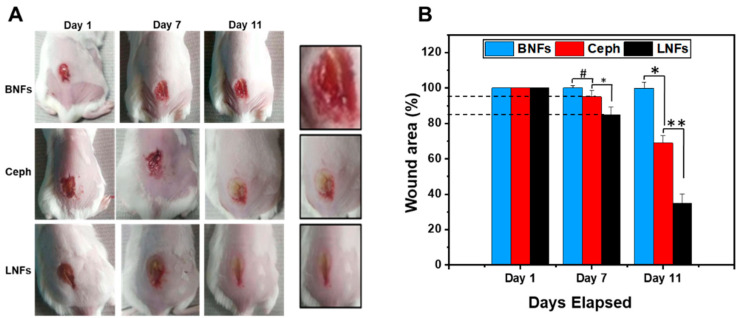
Wound healing capacity of LNFs in the diabetic/obese NcZ10 mouse model. (**A**) One single *S. aureus*-infected wound was created by excision before daily topical treatment with LNFs for 11 days. Ceph and BNFs were used as controls; (**B**) Graphical representation of wound area closure (%) over time in the three groups of mice. Data are expressed as the mean ± SD. ** *p* < 0.01, * *p* < 0.05 are differences considered statistically significant, and ^#^ *p* > 0.05 is considered statistically insignificant.
